# Anti-hepatitis C virus seroprevalence in the working age population in Poland, 2004 to 2014

**DOI:** 10.2807/1560-7917.ES.2017.22.2.30441

**Published:** 2017-01-12

**Authors:** Bożena Walewska-Zielecka, Urszula Religioni, Grzegorz Juszczyk, Zbigniew M Wawrzyniak, Aleksandra Czerw, Piotr Soszyński, Adam Fronczak

**Affiliations:** 1 Department of Public Health, Medical University of Warsaw, Poland; 2 Medicover Sp. z o.o., Poland; 3 Department of Prevention of Environmental Hazards and Allergology, Medical University of Warsaw, Poland; 4 Faculty of Electronics and Information Technology, Warsaw University of Technology, Poland

**Keywords:** hepatitis C virus, HCV infection, anti-HCV

## Abstract

Hepatitis C virus (HCV) infection is considered by the World Health Organization (WHO) to be a serious public health concern and one of the major public health priorities. In 2005, it was estimated that there are 185 million anti-HCV positive people in the world, which constitutes 2.8% of the global population. Our study estimates the anti-HCV seroprevalence in the working age population (15–64 years-old), mostly urban and suburban residents, in Poland from 2004 to 2014. The studied group consisted of 61,805 working-age population representatives whose data were obtained from electronic medical records of an outpatient clinic network operating on a countrywide level. Positive anti-HCV test results were obtained in 957 patients, representing 1.5% of the whole population studied throughout the analysed period. The average age of all anti-HCV positive patients was 36.8 years. Analysis of the data suggests that the proportion of anti-HCV positive patients decreased over the study period (mean positive anti-HCV = -0.0017 × year + 3.3715; R^2^ = 0.7558). In 2004, positive results were noted among 3.2% of patients undergoing HCV antibody tests, but in 2014, the percentage of patients with a positive result stood at 1.1%. The apparent decrease affected men and women similarly. Our study also provides evidence that screening people born before 1965 could be beneficial.

## Introduction

Liver cirrhosis, liver failure and hepatocellular carcinoma are possible long-term consequences of untreated hepatitis C virus (HCV) infection [1-5], which the World Health Organization (WHO) considers as a serious public health concern and one of the major public health priorities [6]. HCV is transmitted mostly by percutaneous exposure to blood [7,8], including intravenous drug injection, which is becoming an important route, especially in developed countries [9,10]. Mother-to-child transmission occurs as well; however, it is relatively uncommon, affecting an estimated 4% of children of HCV-infected mothers [11,12].

In 2005, ca 185 million people in the world, corresponding to approximately 2.8% of the global population, were estimated to be anti-HCV positive [13]. The prevalence of HCV infection ranges from 1.2% to 3.8% in different parts of the world and is highest in central Asia (3.8%), east Asia (3.7%) and North Africa/Middle East (3.6%) [14,15]. In the United States (US), HCV infection prevalence is at 1.6% (2.1% in men and 1.2 % in women) and higher (75% of all cases) in people born between 1945 and 1965 [16]. For this reason, both the Centers for Disease Control and Prevention (CDC) as well as the American Gastroenterology Association (AGA) recommend screening for all individuals born in this period [17,18].

A study from 2014, based on comprehensive literature search anti-HCV prevalence, found the prevalence in Europe to vary from 0.9% in western Europe, through 1.3% in central Europe to 3.3% in eastern Europe [19]. A report from the European Centre for Disease Control and Prevention estimates that in European Union (EU)/European Free Trade Association (EFTA) countries over half of persons with HCV infection in 2006 are in the 25–44 year age group and overall men (64.4%) are more affected than women (35.6%) [20]. From the 1990s up to 2007, new infections appeared to decline in western Europe, while they increased in eastern Europe, possibly due to rising numbers of people who inject drugs (PWIDs) in the east and effective needle sharing programmes in the west [15,21,22].

In Poland, newly diagnosed HCV infections are registered and monitored by the National Institute of Public Health since 1997 [23,24]. The data are based on formal notifications from local Sanitary Inspectorates of newly diagnosed HCV infections according to the national case definition [25]. A regulation of the Minister of Health of 20 September 2012 made anti-HCV tests mandatory in all pregnant women from that year onwards [26].

The latest estimates for HCV infection incidence in the country are 7.99 newly diagnosed cases per 100,000 inhabitants in 2014 [27] and, preliminarily, 11.14 newly diagnosed cases per 100,000 inhabitants in 2015 [24]. HCV infection incidence is much higher in the cities (10.7/100,000 inhabitants) than in rural areas (4.82/100,000 inhabitants) and in men (8.58/100,000) than in women (7.44/100,000) [27]. As acute HCV infection is usually asymptomatic, 86% of infected people in Poland are estimated to be unaware of their infection [14,28]. Therefore increasing the diagnosis rate of infected persons is important [2], not only to more timely treat hepatitis C, but also to stop further spread of HCV. 

Research on HCV prevalence in Poland has so far mainly focused on specific groups (healthcare workers, patients, volunteers, students, blood donors, pregnant women) or on selected areas of Poland [28-36]. There are no epidemiological data for the prevalence of HCV in the general working age population over the whole country, especially based on a large population sample. The purpose of this study is therefore to estimate the anti-HCV seroprevalence in the working age population of Poland, using real-life data obtained from medical records of countrywide outpatient clinics, and accordingly formulate recommendations on age-related HCV infection screening.

## Methods

### Data source

Data were obtained in February 2015 from electronic medical records of a large countrywide outpatient clinic network operating mainly in big cities (with more than 300,000 inhabitants) representing the capitals of 11 of the 16 regions in Poland (Bialystok, Bydgoszcz, Gdańsk, Katowice, Krakow, Lublin, Łódź, Poznań, Szczecin, Warszawa, Wrocław). The clinics provide medical services predominantly to urban and suburban inhabitants with a negligible share of patients from rural areas. It is estimated that study clinics are accessible to a total of 6 million city dwellers (15% of the Polish population).

### Testing for hepatitis C virus antibodies

In order to estimate the seroprevalence in the study population, only the results of anti-HCV were analysed. Anti-HCV in serum was detected by electrochemiluminescence (Roche, ECLIA) and the detection method did not change throughout the study period. All patients with positive results had been referred to special infectious disease clinical departments in order to undergo confirmatory HCV RNA tests if necessary; therefore, those results were not available in the anonymous dataset. Anti-HCV true positive results were not confirmed by immunoblotting. Such a limited approach without final confirmation of anti-HCV positivity was applied on the basis of the European Association for the Study of the Liver (EASL) recommendations, stating that immunoblotting is not recommended to distinguish false positive and true positive anti-HCV result. In order to confirm current viraemia, an HCV RNA test ought to be performed, however this was not the aim of this study [37].

The clinical sensitivity of the test used to detect anti-HCV is estimated at 100% (95% confidence interval (CI): 99.61–100%), the specificity at 99.62% (95%CI: 99.71%–99.92%) [38].

### Study population

The total population aimed to be investigated in the study consisted of patients who had been tested for anti**-**HCV at least once in the period from 2004 to 2014. The study group was extracted from the pool of all medical records of 1.5 million individuals who had been consulted by any doctor in this period. Available records included information on: unique patient number, sex, date of test, age at the date of testing, diagnosis related to the test referral and test result. Only the latest result of testing was included into the study pool, which finally comprised 61,805 single test results of unique patients. The study group was limited to working age population representatives, aged 15–64 years. The working age population was defined according to the definition of the Organisation for Economic Co-operation and Development (OECD) [39]. In Poland, the working age population consists of 25 million people including 11.768 million women and 12.971 million men.

### Data analysis

#### Analysis of the population tested for hepatitis C virus antibodies

The total population tested for anti-HCV was divided into 10-year age groups stratified by sex and the year of testing for time analysis. In each subgroup, the total number of patients tested was used as a denominator.

#### Analysis of the population testing positive for hepatitis C virus antibodies

The number of persons with a positive result for HCV antibody were available each year along with demographical data (sex and age). The rate of total positive tests was calculated and stratified by sex and age. The analyses by age group were conducted by comparing the number of patients, the number of tests and the number of positive/negative results for HCV antibody. Two classifications according to age were used. In the first classification the study population age range was split into 10 year-age groups. In the second classification, since the US data indicated a higher prevalence of HCV in people now aged 50 to 70 years [16], the percentage of positive anti-HCV test results was accordingly analysed in age groups 15 to 49 years and over 50 years. 

The mean rates of positive patients were analysed over time by regression analysis and stratified by sex.

#### Analysis of testing and positive tests by referral group

A number of referrals for anti-HCV test (n = 36,356) had preliminary diagnosis information (according to ICD-10 coding) [40]. We compiled those diagnoses into specific groups for further analyses (Table 1). Both 3-digital and 5-digital ICD-10 codes were aggregated. 

**Table 1:** Aggregated ICD-10 diagnoses accompanying referrals for testing hepatitis C virus antibodies, Poland, 2004–2014 (n = 36,356 patients)

**Diagnosis**	**ICD-10- codes**	**Group**
Pregnancy and pregnancy-related conditions	O20, O24, O26, Z32, Z34, Z35	1
Preventive consultations of generally healthy persons	Z00, Z01, Z02, Z10, Z24, Z29, Z31, Z71, Z76	2
Various symptoms and signs	R10, R53, R68, R69, R72, R79, Z03, Z04	3
Fatty liver disease	K76, E78	4
Hypertransaminasaemia	R74	5
Others	Other than the above	6

### Statistical methods

Data were analysed using STATISTICA (data analysis software system), version 12, (www.statsoft.com) StatSoft, Inc. (2014) US, to calculate the incidence of newly diagnosed cases per year, and the prevalence in the entire examined population. The independent-sample t-test was used for normally distributed variables, and the nonparametric Mann–Whitney U test was used for not normally distributed parameters. Significance was set at p < 0.05. Using linear regression analysis, the trend of the number of the incidence as a function of time (years) was calculated and the R-square value evaluated the goodness of fit of the regression.

## Results

### Characteristics of the study population

#### Overall characteristics

A total of 61,805 single patient records were considered in the study, spanning the period from 2004 to 2014 (Table 2). Men (n = 19,531) accounted for 31.6% of the total study group. The overall average age of patients was 34.4 years (standard deviation (SD): 8.6). The average age of men was 36.5 years (SD: 9.6). The average age of women was 33.4 years (SD: 7.9) (Table 3).

**Table 2:** Annual numbers of patients tested for hepatitis C virus (HCV) antibodies and proportions testing positive, stratified by sex, Poland, 2004–2014 (n = 61,805 patients)

Year	All patients			Women			Men			P value
	Number of anti-HCV tests	Number of positive results	Percentage of positive results	Number of anti-HCV tests	Number of positive results	Percentage of positive results	Number of anti-HCV tests	Number of positive results	Percentage of positive results	
2004	815	26	3.2%	410	14	3.4%	405	12	3.0%	0.7143
2005	1,366	32	2.3%	766	14	1.8%	600	18	3.0%	0.1553
2006	1,210	29	2.4%	650	13	2.0%	560	16	2.9%	0.3314
2007	1,761	43	2.4%	961	26	2.7%	800	17	2.1%	0.4322
2008	3,033	47	1.6%	1,648	25	1.5%	1,385	22	1.6%	0.8740
2009	4,263	75	1.8%	2,477	44	1.8%	1,786	31	1.7%	0.9208
2010	5,250	116	2.2%	3,243	57	1.8%	2,007	59	2.9%	0.0075
2011	7,378	149	2.0%	4,970	86	1.7%	2,408	63	2.6%	0.0180
2012	9,527	133	1.4%	6,730	78	1.2%	2,797	55	2.0%	0.0059
2013	12,239	141	1.2%	8,799	88	1.0%	3,440	53	1.5%	0.0216
2014	14,963	166	1.1%	11,620	113	1.0%	3,343	53	1.6%	0.0090
Total	61,805	957	1.5%	42,274	558	1.3%	19,531	399	2.0%	0.0001

**Table 3:** Characteristics of the study population and that testing positive for hepatitis C virus antibodies, Poland, 2004–2014 (n = 61,805 patients)

Participants	Sex	Mean age	Standard deviation	Number of persons	Column percentage
Study participants	F	33.4	7.9	42,274	68.4%
	M	36.5	9.6	19,531	31.6%
	Total	34.4	8.6	61,805	100.0%
Study participants testing positive	F	36.0	9.8	558	58.3%
	M	37.8	9.7	399	41.7%
	Total	36.8	9.8	957	100.0%

F: female; M: male.

#### Analysis by age group

The most represented age group in terms of number of individuals was the one comprising 25 to 34 year-olds (n = 35,047 patients; 56.7%) and the least numerous group comprised persons over 55 years (n = 2,626 patients; 4.2%) (Table 4).

**Table 4:** Results of anti-hepatitis C virus tests stratified by patient age groups and sex in a study estimating hepatitis C seroprevalence, Poland, 2004–2014 (n = 61,805 patients)

Age group (years)	All patients			Women			Men			P value
	Number	Number testing positive for HCV antibodies	Percentage testing positive for HCV antibodies	Number	Number testing positive for HCV antibodies	Percentage testing positive for HCV antibodies	Number	Number testing positive for HCV antibodies	Percentage testing positive for HCV antibodies	
15–24	3,411	52	1.5%	2,234	33	1.5%	1,177	19	1.6%	0.7561
25–34	35,047	436	1.2%	26,632	285	1.1%	8,416	151	1.8%	0.0001
35–44	15,614	254	1.6%	9,444	132	1.4%	6,170	122	2.0%	0.0051
45–54	5,107	147	2.9%	2,591	65	2.6%	2,536	82	3.2%	0.1318
55–64	2,626	68	2.6%	1,394	43	3.2%	1,232	25	2.0%	0.0893

15–49	56,921	825	1.4%	39,676	480	1.2%	17,245	345	2.0%	0.0001
50–64	4,884	132	2.7%	2,598	78	3.0%	2,286	54	2.4%	0.1687
**Total**	61,805	957	1.5%	42,274	558	1.3%	19,531	399	2.0%	0.0001
Mean age in years (SD)	34.4 (8.6)	36.8 (9.8)	100.0%	33.4 (7.9)	36.0 (9.8)	58.3%	36.5 (9.6)	37.8 (9.7)	41.7%	0.0001

HCV: hepatitis C virus; SD: standard deviation.

#### Time analysis of testing practices

The number of patients examined for anti-HCV increased steadily with time, from 815 patients in 2004, to 14,963 in 2014 (Table 2).

The percentage of all medical-facility-patients tested yearly increased from 0.9% (815/88,177) in 2004 to 4.0% (14,963/376,637) in 2014. Data showed a growing proportion of women being examined. In 2004, a similar number of men and women underwent anti-HCV tests (50.3% (410/815) of women and 49.7% (405/815) of men), whereas in 2014, women accounted for 79.1% (11,620/14,693). This increase may reflect legal requirements for prenatal care during pregnancy in Poland, with HCV testing becoming compulsory for pregnant women from 2012 onwards (Table 5) [26].

**Table 5:** Proportions of patients undergoing anti-hepatitis C virus tests, Poland, 2004–2014 (n = 61,805 patients)

Year	All patients			Women			Men		
	Number	Number tested	Percentage tested	Number	Number tested	Percentage tested	Number	Number tested	Percentage tested
2004	88,177	815	0.9%	45,417	410	0.9%	42,760	405	0.9%
2005	106,464	1,366	1.3%	54,484	766	1.4%	51,980	600	1.2%
2006	127,195	1,210	1.0%	64,518	650	1.0%	62,677	560	0.9%
2007	157,238	1,761	1.1%	80,478	961	1.2%	76,760	800	1.0%
2008	200,031	3,033	1.5%	103,257	1,648	1.6%	96,774	1,385	1.4%
2009	219,905	4,263	1.9%	114,630	2,477	2.2%	105,275	1,786	1.7%
2010	240,307	5,250	2.2%	125,217	3,243	2.6%	115,090	2,007	1.7%
2011	269,140	7,378	2.7%	139,882	4,970	3.6%	129,258	2,408	1.9%
2012	303,813	9,527	3.1%	157,812	6,730	4.3%	146,001	2,797	1.9%
2013	335,526	12,239	3.6%	173,902	8,799	5.1%	161,624	3,440	2.1%
2014	376,637	14,963	4.0%	195,787	11,620	5.9%	180,850	3,343	1.8%
Total	2,424,433	61,805	2.5%	1,255,384	42,274	3.4%	1,169,049	19,531	1.7%

### Characteristics of the population testing positive for hepatitis C virus antibodies

#### Overall characteristics

Throughout the analysed period, 1.5% patients (957/61,805) undergoing anti-HCV examination tested positive. Averaged positive results for women and men were 1.3% (558/42,274) and 2.0% (399/19,531) respectively (p = 0.0001). The average age of all anti-HCV positive patients was 36.8 years (SD: 9.8). The average age of anti-HCV positive women was 36.0 years (SD: 9.8), and the average age of men with positive test results was 37.8 years (SD: 9.7).

#### Analysis by age group

Most anti-HCV positive cases occurred in patients aged 45–54 years (2.9% of tested patients 147/5,107) and in patients older than 55 years (2.6% of tested patients 68/2,626). The lowest proportions of positive test results were noted in the youngest patients: 1.2% (436/35,047) among patients aged 25 to 34 years and 1.5% (52/3,411) among patients aged 15 to 24 years.

In the group of tested women, among those older than 25 years, the percentage of positive anti-HCV test results increased with age, being lowest among women aged 25–34 years (1.1%; 285/26,632) and 35–44 years (1.4%; 132/9,444), and highest for women aged over 55 years (3.2%; 43/1,394). For the youngest group comprising 15 to 24 year-olds, the value 1.5% (33/2,234) was similar to that of the group of 35 to 44 year-olds (1.4%; 132/9,444). In the group of tested men, the smallest proportion of infections was found in the 15 to 24 years age group (1.6%; 19/1,177) and increased with age up to 3.2% (82/2,536) in the age group including 45 to 54 year-olds. For individuals over 55 years the value was similar (2.0%; 25/1,232) to that of the age group with 35 to 44 year-olds (2.0%; 122/6,170) (Table 3).

Because a higher prevalence of HCV was reported in 50 to 70 year-olds in the US [16], the percentage of positive anti-HCV test results was also analysed in age groups 15 to 49 years (representing 92.1% of those tested 56,921/61,805) and over 50 years (7.9%; 4,884/61,805). A higher percentage of anti-HCV positive patients was found in those aged over 50 years (2.7%; 132/4,884) compared with younger participants (1.4%; 825/56,921) (p < 0.0001). This percentage was higher for both women and men aged over 50 years, with, in women 3.0% (78/2,598) vs 1.2% (480/39,676) in those aged under 50 years (p = 0.0001) and, in men, 2.4% (54/2,286) vs 2.0% (345/17,245) in those less than 50 years-old (p = 0.2507).

#### Time analysis of patients testing positive for hepatitis C virus antibodies

An analysis of the data in the years 2004 to 2014 suggests a downward trend for the proportion of positive anti-HCV results (mean positive anti**-**HCV = **-**0.0017 × year + 3.3715; R^2^ = 0.7558) (Figure).

**Figure:**
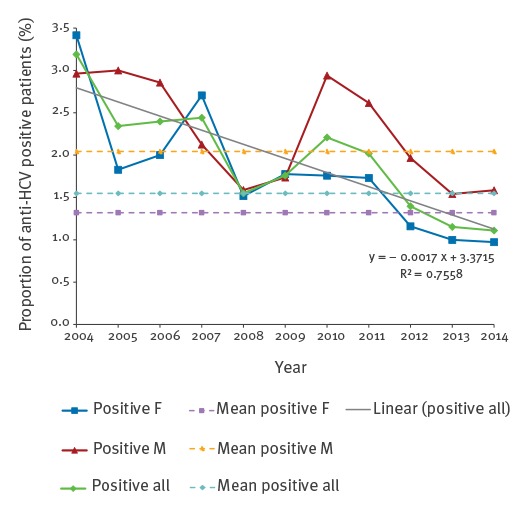
Proportions of hepatitis C virus antibody positive tests among the study population, stratified by year and sex, Poland, 2004–2014 (n = 61,805 patients)

F: female; M: male; HCV: hepatitis C virus.

In 2004, positive results were noted in 3.2% (26/815) of patients examined for anti-HCV, but in 2014 the percentage of patients with a positive result stood at 1.1% (166/14,693). Similar tendencies were observed in both women and men. In 2004, the percentage of anti-HCV positive results in women was 3.4% (14/410), and in men 3.0% (12/405) (p > 0.05), whereas in 2014, anti-HCV positive results were noted among 1.0% (113/11,620) of women and 1.6% (53/3,343) of men (p = 0.0090).

### Referral group testing and test results

We analysed the diagnoses ascribed to each anti-HCV test referral. The predominant reason for anti-HCV testing was pregnancy and pregnancy-related conditions – 44.4% (16,130/36,356) –, followed by preventive testing of otherwise healthy people (occupational health or preventive screening) with 17.8% (6,456/36,356). Various symptoms and signs were the reason for testing in 14.2% of patients (5,151/36,356), fatty liver disease in 3.1% (1,115/36,356) and elevated alanine transaminase levels (ALT) as single diagnosis in 3.0% (1,093/36,356) (Table 6). Only 0.8% (122/16,130) of patients with a diagnosis of pregnancy and pregnancy-related conditions were anti-HCV positive. Preventive action and anti-HCV testing revealed positive results in 1.2% (75/6,456), of patients, while 2.1% (108/5,151) of patients with various symptoms and signs were anti-HCV positive, as well as 3.4% (23/1,115) of patients with a diagnosis of fatty liver disease and 3.7% (25/1,093) of people with elevated ALT (Table 6).

**Table 6:** Primary diagnoses resulting in the referral for anti-hepatitis C virus tests, Poland, 2004–2014 (n = 36,356 patients)

**LBID012_Diagnosis**	**Number of patients tested**	**Number of patients with positive results**	**Proportion of positive patients **	**Proportion of positive** **women**	**Proportion of positive men**	**Proportion positive in column**	**Proportion of diagnosed patients**
Pregnancy and pregnancy related conditions	16,130	122	0.8%	0.8%	NA	25.4%	44.4%
Preventive consultations of generally healthy persons^a^	6,456	75	1.2%	0.7%	1.0%	15.6%	17.8%
Various symptoms and signs	5,151	108	2.1%	1.0%	2.1%	22.5%	14.2%
Hypertransaminasaemia^b^	1,093	25	3.7%	1.1%	1.7%	5.2%	3.0%
Fatty liver disease	1,115	23	3.4%	0.8%	1.7%	4.8%	3.1%
Others^c^	6,411	127	1.5%	0.9%	2.6%	26.5%	17.6%
Total	36,356	480	1.1%	0.8%	1.9%	100.00%	100.0%

NA: not applicable.

^a^ Spontaneous or obligatory health check-ups.

^b^ Excluding people with elevated transaminase level as a reason of additional anti-HCV test.

^c^ Most often before surgical procedures.

## Discussion

Our study presents an evaluation of anti-HCV prevalence in a large country-wide sample of (sub)urban working-age Polish people between 2004 and 2014. Data from electronic medical records of ambulatory patients visiting doctors due to different conditions, including prophylactic and screening reasons, were analysed. The overall anti-HCV prevalence in our study was 1.5%. Similar to previous studies [29,30] and studies from other countries [41-43], anti-HCV positivity was significantly more frequent in men than women (2.0% vs 1.3% respectively; p = 0.0001). 

We also found that in contrast to younger age groups (15 to 49 years), anti-HCV prevalence in people aged between 50 and 64 years was higher (2.7% vs 1.4%; p < 0.0001), and surprisingly more frequent in women (3.0%) than men (2.4%) although, this difference was not statistically significant. Higher HCV infection prevalence in people born before 1965 has also been observed in the US [16], therefore, the recommendation of CDC and AGA to screen people born before 1965 [17,18] might also be justified in Poland and could be implemented as part of primary healthcare. According to the authors’ own research on 16,130 pregnant women, the prevalence of HCV in this group was only 0.8% which is close to the European average (1%) [44]. Low anti-HCV prevalence in pregnant women and high prevalence in people of post-reproductive age might be a subject of debate in terms of allocating effective financial resources for HCV screening in these two groups.

To our knowledge, our study constitutes one of the currently largest performed in the Polish population of working age. Indeed, although previous studies in the country have attempted to assess HCV prevalence, these have either been conducted either some time ago, or have been mainly based on small samples and/or on selected population groups – pregnant women, students, blood donors or deceased organ donors – with, in some cases, only a single district or town in the country considered [28,29,31-35,45]. For example, the study by Bielawski et al. in 1999, which is still the point of reference for many epidemiological studies on HCV infection in Poland, was conducted on a group of 2,561 volunteers enrolled by a laboratory in Gdansk in response to press advertisements. It estimated the overall HCV infection prevalence at 1.9% [29], with 2.3% of men versus 1.7% of women seropositive for hepatitis C virus antibodies. Limitations of this study were however the restricted geographical area and the group chosen to be tested (volunteer bias) [29]. Since then, larger studies have been performed, an important one being that of Seyfried et al., where 4,233,119 blood donors were screened between 1994 and 2003. Anti-HCV prevalence in this group was found to be on average 0.5% [36]. The most recent study by Flisiak et al. in 2011, which was performed from 2009 to 2010 in 26,057 Polish adults (healthcare workers and hospital patients), presented anti-HCV prevalence in healthcare workers at 1.4%, whereas in ambulatory patients of different general practice and specialist outpatient clinics it was 1.9% [30]. Here we investigate 61,805 people of working-age over the whole country between 2004 and 2014 and find a prevalence of 1.5%, similar to what is currently estimated for central Europe (1.3%) [19]. 

Hepatitis C infection has been registered as a distinct disease entity in Poland since 1997. Until 2004, the annual number of newly diagnosed cases of HCV infection, logged by the National Institute of Hygiene, did not exceed 2,000. In the years 2005 and 2006 the number increased to 3,000 (2,997 and 2,949 in each year respectively), but this increase was most likely caused by the modification of disease reporting methods. In the following years, the number of newly diagnosed cases decreased steadily (2,753 in 2007, 2,353 in 2008). Since 2009 however, another upward trend can be observed. The number of reported cases of new HCV infections in Poland accumulated to 1,891 in 2009, 2,178 in 2010, 2,189 in 2011, 2,265 in 2012 and increased to 2,600 cases in 2013 [46]. 

Our analysis on the proportions of persons with HCV antibodies in working age people from 2004 to 2014 does not find such an increasing trend in the latest years of the study. Instead we find a higher proportion of patients with HCV antibodies in the first year of the study compared to the end of the analysed period (3.2% in 2004 vs 1.1% in 2014). This could be due to a general drop in HCV infections in working age people, but also to a change of indication for testing. Indeed, the most common indication for evaluating HCV serological status in the early years of the study was elevated serum ALT, which per se is regarded as a laboratory manifestation of liver injury. With the introduction of obligatory anti-HCV testing in all pregnant women from 2012 onwards, the proportion of anti-HCV positive persons dropped significantly, possibly coming closer to the actual prevalence of HCV infection in this relatively young and overall healthy group.

This study presents however a number of limitations. First, the prevalence of HCV infection is known to vary according to risk groups. The study by Flisiak et al. on 17,930 persons found that significant factors of HCV infection in Poland are more than three hospitalisations during a life time (odds ratio (OR) = 1.8), blood transfusion before 1992 (OR = 2.9) and intravenous drug use (OR = 6.2) [30]. Transmission of HCV via intravenous drug use has been increasingly observed and in 2007, 10 of 16 million of PWID worldwide were estimated to be HCV positive. The number of active PWIDs in the EU is estimated at ca 1 million [10]. In Poland, 70% of PWIDs are infected with HCV, predominantly men under 45 years of age [47]. In our study, we had no access to individual medical records; therefore, intravenous drug use could not be accounted for. Moreover, we did not have information on patients’ profession either, so we could not evaluate any possible occupational risk. 

Second, our study only analysed HCV antibody prevalence, and western blot tests were not performed. Thus results do not distinguish between current infections and probable infections in the past (resolved infection). A final diagnosis of current HCV infection requires the finding of HCV RNA in serum samples by RT-PCR. We had no access to HCV RNA results that were stored in the form of scans and required patients’ consent for access. The results of this study can therefore not be compared with any studies using the EU HCV case definition [48]. A Polish study in 2011 showed that only 31% of those with HCV antibodies were also positive for HCV RNA by RT-PCR [30]. Moreover the sensitivity of the electrochemiluminescence (Roche, ECLIA) assay used in this study is very high, so specificity will be lower, which may result in false positive results. Taking these points into consideration, the prevalence of current HCV infection in the Polish urban working population is likely to be lower than 1.5%. Further studies on positive anti-HCV test results and HCV RNA detection may reveal if HCV infection is resolved more frequently than has been presumed up to now (ca 30% of cases being resolved [49]).

Finally, although our study was conducted on a large number of patients, another important limitation was the inclusion of only people living in big cities and their suburbs. One risk factor for HCV infection is the use of medical care, which is less frequent in inhabitants of rural areas. Accordingly, anti-HCV prevalence in rural areas has been shown to be lower than in urban areas [27,50]. Therefore our results cannot be extrapolated to the whole population and it can be assumed that the prevalence of HCV infection among people of working age in the country may be lower than 1.5%.

## Conclusions

There is evidence that an improvement of diagnostics and treatment effectiveness may significantly reduce the burden of HCV infections in Poland [5,51]. A study using a modelling approach estimated that, until 2030, the HCV prevalence is projected to decrease by 5%. In contrast, an increase in the number of treated patients to 15,000 yearly would reduce the number of total infections by 90% until 2030, which would also contribute to a decrease of HCV-mortality by 80% [49]. The results obtained in this study suggest that the proportion of people infected with HCV in Poland in the working population is decreasing, which may be a consequence of increasing social awareness, including preventative activities after or before exposure to blood-borne infections. Moreover, a higher prevalence of anti-HCV was found in the population of post-reproductive age. We therefore recommend screening HCV tests mainly in individuals over 45 years-old. Examining healthy and young people should not be carried out as part of screening, however testing may be recommended to individuals who are subjected to risk factors. The continuous monitoring of HCV prevalence and incidence in Poland is important to estimate the resources needed for screening and treatment as well as their costs. Knowing the age groups at higher risk for infection will help to establish recommendations for more effective detection of cases of HCV infection, which in turn is also crucial to reduce further transmission.
